# Simplified antibiotic regimens for treatment of clinical severe infection in the outpatient setting when referral is not possible for young infants in Pakistan (Simplified Antibiotic Therapy Trial [SATT]): a randomised, open-label, equivalence trial

**DOI:** 10.1016/S2214-109X(16)30335-7

**Published:** 2016-12-15

**Authors:** Fatima Mir, Imran Nisar, Shiyam S Tikmani, Benazir Baloch, Sadia Shakoor, Fyezah Jehan, Imran Ahmed, Simon Cousens, Anita K M Zaidi

**Affiliations:** a Department of Pediatrics and Child Health, Aga Khan University, Karachi, Pakistan; b Department of Microbiology, Aga Khan University, Karachi, Pakistan; c Data Management Unit, Aga Khan University, Karachi, Pakistan; d London School of Hygiene & Tropical Medicine, London, UK; e Enteric and Diarrheal Diseases, Global Health, Bill & Melinda Gates Foundation, Seattle, WA, USA

## Abstract

**Background:**

Parenteral antibiotic therapy for young infants (aged 0–59 days) with suspected sepsis is sometimes not available or feasible in countries with high neonatal mortality. Outpatient treatment could save lives in such settings. We aimed to assess the equivalence of two simplified antibiotic regimens, comprising fewer injections and oral rather than parenteral administration, compared with a reference treatment for young infants with clinical severe infection.

**Methods:**

We undertook the Simplified Antibiotic Therapy Trial (SATT), a three-arm, randomised, open-label, equivalence trial in five communities in Karachi, Pakistan. We enrolled young infants (aged 0–59 days) who either presented at a primary health-care clinic or were identified by a community health worker with signs of clinical severe infection. We included infants who were not critically ill and whose family refused admission. We randomly assigned infants to either intramuscular procaine benzylpenicillin and gentamicin once a day for 7 days (reference); oral amoxicillin twice daily and intramuscular gentamicin once a day for 7 days; or intramuscular procaine benzylpenicillin and gentamicin once a day for 2 days followed by oral amoxicillin twice daily for 5 days. The primary outcome was treatment failure within 7 days of enrolment and the primary analysis was per protocol. We judged experimental treatments as efficacious as the reference if the upper bound of the 95% CI for the difference in treatment failure was less than 5·0. This trial is registered at ClinicalTrials.gov, number NCT01027429.

**Findings:**

Between Jan 1, 2010, and Dec 26, 2013, 2780 infants were deemed eligible for the trial, of whom 2453 (88%) were enrolled. Because of inadequate clinical follow-up or treatment adherence, 2251 infants were included in the per-protocol analysis. 820 infants (747 per protocol) were assigned the reference treatment of procaine benzylpenicillin and gentamicin, 816 (751 per protocol) were allocated amoxicillin and gentamicin, and 817 (753 per protocol) were assigned procaine benzylpenicillin, gentamicin, and amoxicillin. Treatment failure within 7 days of enrolment was reported in 90 (12%) infants who received procaine benzylpenicillin and gentamicin (reference), 76 (10%) of those given amoxicillin and gentamicin (risk difference with reference −1·9, 95% CI −5·1 to 1·3), and 99 (13%) of those treated with procaine benzylpenicillin, gentamicin, and amoxicillin (risk difference with reference 1·1, −2·3 to 4·5).

**Interpretation:**

Two simplified antibiotic regimens requiring fewer injections are equivalent to a reference treatment for young infants with signs of clinical severe infection but without signs of critical illness. The use of these simplified regimens has the potential to increase access to treatment for sick young infants who cannot be referred to hospital.

**Funding:**

The Saving Newborn Lives initiative of Save the Children, through support from the Bill & Melinda Gates, and by WHO and USAID.

## Introduction

Despite improvements in child survival over recent decades, progress in newborn survival remains slow, with 44% of all child deaths occurring in the first month of life. Of these neonatal deaths, 23–30% are due to infections.^1^

WHO recommends hospital referral and 7 days of injectable penicillin and gentamicin for neonates and young infants (aged 0–59 days) with suspected sepsis.^2^ However, up to three-quarters of families of sick young infants in Karachi, Pakistan, refuse hospital referrals, despite free transport and treatment, because of the substantial opportunity costs to very poor families of prolonged admissions at locations far from their place of residence.^3^ Stated reasons for refusal are financial constraints, cultural beliefs, and concern about poor quality of care at hospitals.^3^, ^4^ Similar constraints to optimum care of sick newborn babies in high-mortality settings have also been noted from other low-resource settings.^5^

An expert consultation reviewed the issue of low adherence to referral advice for sick young infants and recommended that clinical trials were needed to evaluate simplified antibiotic regimens to manage severe infections in young infants when referral was not possible, to improve access to care and newborn survival.^6^ Thus, randomised controlled trials assessing simplified antibiotic regimens—ie, fewer injections, addition of high-dose oral amoxicillin in lieu of penicillin—for outpatient management of young infants with clinical severe infection were undertaken in several countries (Democratic Republic of Congo, Bangladesh, Kenya, Nigeria, and Pakistan) to ensure wide generalisability.^7^, ^8^, ^9^, ^10^ These trials were not designed to show that the simpler regimens were better than the standard regimen but rather that they had similar efficacy to the reference regimen—namely, an equivalence or non-inferiority design.^11^ Thus, the trials were designed to produce narrow confidence intervals when examining the difference in risk of treatment failure between the simplified regimens and the reference treatment and, hence, provide a high degree of confidence that any differences between efficacy of the treatments were small. The choice of treatment regimens followed a systematic review of pathogens causing neonatal sepsis,^12^ their antimicrobial resistance patterns,^13^ antibiotic pharmacodynamics in neonates,^14^ and existing evidence on treatment success with various oral and injectable antibiotics in young infants.^15^, ^16^

**Research in context**

**Evidence before the study**

We searched PubMed between January, 1990, and October, 2015, with the terms “young infant”, “clinical severe infection”, and “simplified antibiotic regimens” to identify peer-reviewed publications in the English language about simplified antibiotic regimens for severe infections in neonates and young infants in the primary-care setting. We identified five reports (three protocol papers), of which two reported findings of similar trials undertaken in Africa (Kenya, Nigeria, and Democratic Republic of Congo) and Bangladesh, comparing the WHO-recommended regimen of parenteral penicillin and gentamicin with simpler antibiotic regimens.

**Added value of this study**

Compared with previous studies in Africa and Bangladesh, our trial from Pakistan had a higher representation of very young infants (those in the first week of life) and was enriched by the availability of bacterial aetiological data and antimicrobial susceptibility data. A pooled analysis can now be done of data from all three related trials, to support policy recommendations for this very young group of patients.

**Implications of all the available evidence**

The findings of our trial are consistent with those published previously, showing that simplified antibiotic regimens are as efficacious as the WHO-recommended regimen of parenteral penicillin and gentamicin for young infants with severe infection. Our study has contributed to development of new WHO guidelines for treatment of severe infection in young infants where referral is not feasible.

We targeted young infants (aged 0–59 days) with clinical severe infections because increased susceptibility to infection persists into the second month of life.^17^ Additionally, the signs and management of sepsis in young infants (aged 29–59 days) are similar to those in neonates (aged 0–28 days). WHO and UNICEF's Integrated Management of Childhood Illness (IMCI) strategy addresses children aged 0–59 days as a separate group (young infants) from children aged 2–59 months.^18^

Here, we present data from the Simplified Antibiotic Therapy Trial (SATT) undertaken in Karachi, Pakistan,^8^ to compare the risk of treatment failure in young infants with a diagnosis of clinical severe infection between a reference treatment and two simplified antibiotic regimens, comprising fewer injections and a high dose of an oral antibiotic in lieu of parenteral administration.

## Methods

### Study design and participants

SATT Pakistan is a randomised open-label trial designed to assess equivalence of three outpatient-based antibiotic regimens. We undertook the trial in five low-income settlements in coastal Karachi, Pakistan (Rehri Goth, Ibrahim Hyderi, Ali Akbar Shah Goth, Bhains colony, and Bilal colony), which are roughly 1 h drive from the main campus of the Aga Khan University in Karachi. These settlements are served by five primary health-care clinics, have an annual birth cohort of 8000, and have ongoing household surveillance of young infants aged 0–59 days (appendix p 1).

Infants from the catchment area were either referred to a study clinic by community health workers during routine household surveillance or presented with their family at one of the five primary health-care clinics, at which study clinicians screened them for eligibility to participate in the trial.^8^ Inclusion criteria were age 0–59 days, living in the catchment area, refusal by family to be admitted to hospital, and one or more signs of clinical severe infection (panel). Infants were excluded from the study if their family agreed to admission, weight at presentation was less than 1500 g, major congenital malformations or suspected chromosomal abnormalities were present, surgical conditions needed hospital referral, they had been admitted for illness in the past 2 weeks, they had been included previously in the study, or they had one or more signs of critical illness (panel). If the infant had signs of clinical severe infection, the study clinician first recommended hospital referral. If the family refused admission and the infant had no signs of critical illness, the study clinician offered trial enrolment with facility-based and home-based treatment. If the infant had signs of critical illness, parents were counselled again on the importance of hospital referral. If parents still refused admission, they were offered the reference treatment (ie, procaine benzylpenicillin and gentamicin) and were not enrolled or randomly allocated. Study methods have been described in detail.^8^

We obtained written informed consent for infants to participate in the trial from parents or guardians. Consent included documentation of the refusal for hospital referral and acceptance of enrolment. A third party (community member) witnessed the consent procedure, and this individual also signed the consent form. We read the consent form to illiterate participants and took a thumbprint in lieu of a signature, which a third party witness countersigned. The study was approved by the ethics review committee of the Aga Khan University, ethics review committee of WHO, and the ethics committee at the London School of Hygiene & Tropical Medicine.

### Randomisation and masking

After obtaining consent, we randomly allocated infants to one of three antibiotic regimens: 7 days of procaine benzylpenicillin and gentamicin, administered intramuscularly (reference treatment); intramuscular gentamicin once a day and oral amoxicillin twice daily for 7 days; or procaine benzylpenicillin and gentamicin administered intramuscularly once a day for 2 days followed by oral amoxicillin twice daily for 5 days. We used a site-specific and age-specific (<7 days and 7–59 days) randomisation sequence list generated by the London School of Hygiene & Tropical Medicine.^8^ The allocation sequence for every site and age group was placed in serially numbered, sealed, opaque envelopes by the Data Management Unit at Aga Khan University and delivered to every site. Study clinicians selected the next envelope and the treatment corresponding to the allocation code printed within was assigned to the infant. Study participants' families and study clinicians were not blinded to treatment allocation because giving placebo injections to sick young infants was judged unethical.

### Procedures

Study drugs were provided by the Aga Khan University pharmacy, stored at room temperature away from direct sunlight at study clinics, and administered using study-specific dosage charts present at every public health-care clinic (appendix p 2). We administered intramuscular procaine benzylpenicillin (40 000–60 000 units per kg) once a day, intramuscular gentamicin (4·0–5·0 mg/kg in early neonates aged 0–6 days; 5·0–6·5 mg/kg in infants aged 7–59 days) once a day, and oral amoxicillin (75–100 mg/kg per day) twice daily in divided doses. Paramedics or study clinicians administered intramuscular injections at study clinics; study personnel gave the morning dose of oral amoxicillin at the clinic, and a community health worker visiting the child's household administered the evening dose. If the baby vomited within 30 min, the oral drug was re-administered. We followed up every participant daily at the public health-care clinics, from enrolment to day 8, then on day 11 and day 14 for vital signs (respiratory rate, temperature, and heart rate), danger signs of critical illness (panel), improvement or deterioration in clinical status (defined as resolution or presence of one danger sign of critical illness),^8^ and adverse events (ie, relapse, death, or treatment failure). We referred young infants who failed treatment to hospital. If families still refused admission, we treated the infant with intramuscular ceftriaxone once a day for 1 week.

We collected blood samples at enrolment using appropriate aseptic precautions. We captured blood sample collection procedures on video for about 10% of participants as a quality assurance measure. We injected about 2–3 mL of blood (median 2·1 mL [IQR 1·4–2·8]) into a bottle (BACTEC Peds Plus; Becton Dickinson, Franklin Lakes, NJ, USA) and transported the sample to the Infectious Disease Research Laboratory at Aga Khan University within 3 h of collection; we incubated the sample in a continuous monitoring system (BACTEC 9240; Becton Dickinson) for 5 days. If bottles were flagged positive by the automated system, we Gram-stained and subcultured blood culture broth on appropriate media—eg, 5% sheep blood agar and chocolate agars incubated in 5% CO_2_ at 35°C; Maconkey agar incubated in air at 35°C; or 5% sheep blood agar incubated anaerobically at 35°C. We did all identification and susceptibility tests in accordance with American Society for Microbiology procedures and Clinical Laboratory Standards Institute guidelines,^19^ when applicable. For a few blood cultures that were smear-positive for campylobacter-like organisms but that did not yield bacterial growth on conventional culture, we also did PCR of the 23S RNA-conserved region of campylobacter, helicobacter, and arcobacter complex. Briefly, we extracted DNA (MagNA Pure extraction kit; Roche Diagnostics, Burgess Hill, UK) then did conventional PCR (Eppendorf MasterCycler Gradient; Marshall Scientific, Hampton, NH, USA)^20^ and gel electrophoresis.

We categorised blood culture results as no growth, contamination, or bacteraemia (known or probable pathogen). We classed blood cultures with growth of common skin flora as contamination. We used bacteraemia to describe blood cultures positive for known pathogens, whether isolated singly or mixed with other pathogens or contaminants, or pathogens less commonly associated with neonatal sepsis, whether isolated singly or mixed with other pathogens or contaminants (appendix p 3).

We checked for adverse events at every follow-up visit. Community health workers reported adverse events to study doctors at every study clinic, who graded them as serious or non-serious. Serious adverse events were those possibly related to study drugs, including: severe diarrhoea with dehydration requiring facility management; Stevens-Johnson syndrome; anaphylaxis; and acute renal failure. A study supervisor (trained paediatrician) verified the grading. Quarterly adverse event reports were reviewed by the study's data safety and monitoring board and technical scientific committee.

### Outcomes

The primary outcome of our trial was treatment failure within 7 days of enrolment, which we defined as either: death; admission; clinical deterioration;^8^ change in antibiotic regimen because of infectious comorbidity (to intramuscular ceftriaxone); serious adverse event; occurrence of a new sign of clinical severe infection (panel) on or after day 3; persistence of presenting signs at day 4; or recurrence of initial signs of sepsis on or after day 5. Among young infants who had treatment failure, secondary outcomes were: death within 7 days of enrolment; death at any time before the day 14–15 assessment; and admission for any reason at any time within 7 days of enrolment. Among children who did not have treatment failure, secondary outcomes were: admission at any time between the day 8 and day 14–15 visits; death at any time between the day 8 and day 14–15 visits; and non-fatal relapse at any time between the day 8 and day 14–15 visits (defined as admission, development of any sign of critical illness, or development of any sign of suspected sepsis).

### Statistical analysis

We postulated (from our previous experience) that the simplified antibiotic regimens would have equivalent efficacy to the reference treatment and that the risk of treatment failure would be 10% in all groups. For every comparison with the reference treatment, we planned to estimate the difference in the risk of failure between the two treatment groups and to use a two-sided 95% CI to assess the equivalence of the two simplified antibiotic regimens. We judged simplified antibiotic regimens as efficacious as the reference if the upper bound of the 95% CI for the difference in treatment failure was less than 5. Based on this criterion, we estimated that a sample size of 750 assessable children per treatment group would provide at least 90% power to show that the simplified antibiotic regimens were as efficacious as the reference treatment.

Since the aim of our trial was to show the equivalence of different antibiotic regimens, rather than superiority of one regimen over another, we analysed the primary outcome per protocol^8^ rather than by intention to treat, which would tend to reduce any differences between treatment regimens. We defined per-protocol infants as those who had completed clinical follow-up fully (eight visits on 8 days) or partly (three visits days 2–4, at least one visit days 5–8, and known vital status at day 8) and who had adhered to treatment fully or partly (appendix p 6). We defined infants who were fully adherent to treatment as those who received all doses of scheduled antibiotics for 7 days (or by the time of treatment failure, if failure occurred) and who had not received any other antibiotic from a study or non-study clinician. We defined infants who were partly adherent to treatment as those who had received all scheduled antibiotics on the first 3 days of treatment (or by the time of treatment failure) and at least 50% of all scheduled doses of each antibiotic on days 4–7 (or by the time of treatment failure), and who did not receive any non-study injectable antibiotic before the day 8 visit (unless given because of treatment failure) or any non-study oral antibiotic on days 1–3.

We did statistical analyses with Stata version 13. This study is registered with ClinicalTrials.gov, number NCT01027429.

### Role of the funding source

This trial was funded by the Saving Newborn Lives initiative of Save the Children, with support from the Bill & Melinda Gates Foundation, and by WHO and USAID. The funders had a role in study design but played no part in data collection, data analysis, data interpretation, or writing of the report. The corresponding author had full access to all the data in the study and was responsible for the decision to submit for publication.

## Results

Between Jan 1, 2010, and Dec 26, 2013, 41 230 young infants were screened for trial eligibility and 2780 (7%) were eligible for enrolment (figure). Of these, 63 (2%) families agreed to admission and 264 (9%) refused participation. Thus, 2453 (88%) of 2780 young infants were randomly allocated one of the three study treatments: 820 were assigned procaine benzylpenicillin and gentamicin, 816 were allocated amoxicillin and gentamicin, and 817 were assigned procaine benzylpenicillin, gentamicin, and amoxicillin. Table 1 shows baseline characteristics of all young infants who were randomly allocated. Median age at presentation was 11 days (IQR 2–36). 1083 (44%) infants were early neonates (aged 0–6 days), 1309 (53%) were boys, and 940 (38%) had a low weight-for-age (*Z* score <–2). 2141 (87%) infants presented with one sign at enrolment. Fever was the most common presenting sign (1015/2453 [41%]) and was the only presenting sign for more than a third of infants (905/2453 [37%]; table 1). The next most frequent sign was severe chest indrawing (818/2453 [33%]), with around a third having this sign in isolation (717/2453 [29%]). 358 (15%) of 2453 infants had local infection, of whom 283 (79%) had an umbilical infection and 86 (24%) had a skin infection. 1223 (50%) of 2453 young infants were born in a health facility.

2251 (92%) of 2453 infants who were randomly allocated met per-protocol criteria for clinical follow-up and treatment adherence (figure). 747 received procaine benzylpenicillin and gentamicin, 751 were treated with amoxicillin and gentamicin, and 753 were given procaine benzylpenicillin, gentamicin, and amoxicillin. Per-protocol infants had similar characteristics at baseline to the intention-to-treat population (data not shown). Table 2 presents primary and secondary outcome data in the per-protocol population. Treatment failure was recorded within 7 days of enrolment in 90 (12%) of 747 infants who received procaine benzylpenicillin and gentamicin (reference), 76 (10%) of 751 who were given amoxicillin and gentamicin (risk difference with reference, −1·9, 95% CI −5·1 to 1·3), and 99 (13%) of 753 treated with procaine benzylpenicillin, gentamicin, and amoxicillin (risk difference with reference, 1·1, −2·3 to 4·5); the upper bound of the 95% CI for both comparisons was within the prespecified margin for equivalence. The most common causes of treatment failure across the three study groups were persistence of presenting signs at day 4 (n=62), admission (n=51), and clinical deterioration (n=43). In analyses of all randomly allocated infants (appendix p 4), treatment failure was recorded in 97 (12%) of 820 assigned procaine benzylpenicillin and gentamicin (reference), 81 (10%) of 816 allocated amoxicillin and gentamicin (risk difference with reference, −1·9, 95% CI −4·9 to 1·1), and 111 (14%) of 817 assigned procaine benzylpenicillin, gentamicin, and amoxicillin (risk difference with reference, 1·8, −1·5 to 5·0).

28 (1%) of 2251 infants in the per-protocol analysis died within 7 days of enrolment, due to clinical severe illness, and the risk of death was similar across the three treatment groups (table 2): 11 (1%) of 747 infants died who received procaine benzylpenicillin and gentamicin (reference), seven (1%) of 751 died who were treated with amoxicillin and gentamicin (risk difference with reference −0·5, 95% CI −1·6 to 0·6), and ten (1%) of 753 died who were given procaine benzylpenicillin, gentamicin, and amoxicillin (risk difference −0·1, −1·3 to 1·0). No deaths were attributable to study procedures. A further six children died between day 8 and day 15: two who were treated with procaine benzylpenicillin and gentamicin (both neonatal sepsis); two who received amoxicillin and gentamicin (diarrhoea, and neonatal sepsis); and two who were given procaine benzylpenicillin, gentamicin, and amoxicillin (neonatal tetanus, and neonatal sepsis). None of these six deaths was judged attributable to study procedures by the principal investigator or the data safety and monitoring board. Among all randomly allocated infants, 35 (1%) of 2453 children had died within 7 days of enrolment (appendix p 4), 12 (1%) of 820 children assigned procaine benzylpenicillin and gentamicin (reference), ten (1%) of 816 allocated amoxicillin and gentamicin (risk difference with reference, −0·2, 95% CI −1·4 to 0·9), and 13 (2%) of 817 assigned procaine benzylpenicillin, gentamicin, and amoxicillin (risk difference with reference, 0·1,–1·1 to 1·3). By day 15, a further three infants had died who were allocated procaine benzylpenicillin and gentamicin, two children assigned amoxicillin and gentamicin had died, and three children died who were allocated procaine benzylpenicillin, gentamicin, and amoxicillin.

Three non-fatal serious adverse events were reported among the 2453 randomly allocated infants, one in a child assigned procaine benzylpenicillin and gentamicin (diarrhoea with severe dehydration) and two in children allocated amoxicillin and gentamicin (diarrhoea with severe dehydration, and generalised rash). Two of these three events contributed to the initial reason for treatment failure at day 2. All three infants had recovered by the day 15 visit and were included in the per-protocol analysis. Among the 2251 children in the per-protocol analysis, 193 (9%) non-serious adverse events occurred, 79 (11%) in 747 infants treated with procaine benzylpenicillin and gentamicin, 55 (7%) in 751 who received amoxicillin and gentamicin, and 59 (8%) in 753 who were given procaine benzylpenicillin, gentamicin, and amoxicillin. The most frequent events were injection-site swelling (n=88) and mild diarrhoea (n=81).

Blood cultures were obtained from 2067 (84%) of 2453 randomly allocated infants, of which 1713 (83%) were negative after 5 days of incubation and 273 (13%) were contaminated. 81 (4%) cultures were positive for various pathogens (appendix p 5); 79 grew a single organism whereas two were polymicrobial. Campylobacteraceae were the commonest group of pathogens cultured (n=18), followed by pseudomonads (n=13), enteric Gram-negative bacteria (n=12), and *Streptococcus pyogenes* (n=8). Of 23 Gram-positive organisms, 19 (83%) were susceptible to penicillin. Of the Gram-negative bacteria, gentamicin susceptibility results were available for 14 organisms, and all but one *Klebsiella pneumoniae* isolate were gentamicin susceptible. Ampicillin-susceptibility test results were available for 18 Gram-negative isolates, and nine (50%) were ampicillin susceptible. Overall, 32 (86%) of 37 microbes available for antimicrobial susceptibility were sensitive to a regimen including penicillin or amoxicillin and gentamicin. Ten (13%) of 75 children with bacteraemia and 227 (12%) of 1618 without bacteraemia had treatment failure; thus, bacteraemia did not predict treatment failure in per-protocol infants (risk difference 1·03, 95% CI −6·8 to 8·9).

## Discussion

Our results show that, in Pakistan, simplified antibiotic regimens are as efficacious as the reference treatment for young infants with clinical severe infection whose families refuse referral to hospital.^2^ This finding is consistent with those of two similar trials from Africa,^9^ and Bangladesh.^10^ Results from these trials have contributed to development of new WHO guidelines for management of young infants with possible serious bacterial infection where referral is not feasible.^21^

A strength of our Pakistan study is that we included a much higher representation of infants aged 0–6 days with clinical severe infection (44% of all enrolled children) compared with the other randomised equivalence trials of the simplified antibiotic regimens from Bangladesh^10^ and Africa (Kenya, Nigeria, and Democratic Republic of Congo).^9^ Additional strengths of our study are the availability of bacterial aetiological data and antimicrobial susceptibility data. Enrolment of few infants younger than 7 days with clinical severe infection was judged a limitation of the Bangladesh trial,^22^ in terms of drawing conclusions for this important subgroup at high risk of vertically acquired infections and mortality. A pooled analysis of data from our trial, the Bangladesh trial, and the AFRINEST trial in Kenya, Nigeria, and Democratic Republic of Congo is underway and will have an adequate number of infants aged 0–6 days to support policy recommendations for this age group. These findings hold great promise in increasing access to treatment for sick young infants and improving their clinical outcomes.

Data for common bacterial pathogens among young infants at the community level are scarce.^23^ We gathered samples for blood culture at the first-level facility, from young infants recruited from the community. The proportion of infants with clinical sepsis and who grew a pathogen from the blood sample was low (4%) but comparable with yields reported elsewhere.^24^, ^25^ Biomarkers need to be developed to improve our ability to distinguish between infants with and without bacterial infection in first-level settings.^23^ Bacteraemia did not predict treatment failure in per-protocol infants in our study; however, in view of the fairly small number of culture-positive cases, we cannot exclude a modest overall increase in risk of treatment failure.

Positive blood culture results showed diversity in causative agents of clinical severe infection in young infants. Campylobacteraceae were the commonest pathogens causing bacteraemia and bacteraemic treatment failure. *Campylobacter jejuni* and *Campylobacter coli* are common causes of diarrhoea in infants in developing countries, including the area from which these infants were recruited;^26^ however, none of the young infants in this study who grew campylobacteraceae from their blood had a history of diarrhoea. A related species, *Campylobacter fetus*, has a well known association with adverse birth outcomes in cattle.^27^ It is possible that the fastidious nature and special growth requirements of campylobacteraceae have resulted in scant recognition of its role as a pathogen of newborn bloodstream infections.^28^

One concern was not borne out—namely, that high rates of antimicrobial resistance among newborn pathogens reported from hospital-based series from low-income settings would be a problem for management of clinical severe infection in young infants in the community. Susceptibility testing showed that a regimen based on penicillin or amoxicillin plus gentamicin would cover more than 80% of pathogens encountered.

The mix in severity of clinical severe infections in our study was affected by the proportion of infants identified at a very early stage by household surveillance. However, we made this trade-off to ensure adequate enrolment of infants aged 0–6 days who otherwise do not present to facilities. Another consideration is whether the milder spectrum of illness, potentially including many children without a bacterial infection, could have biased the results of our trial towards equivalence. Although some enrolled children will undoubtedly not have had a bacterial infection, in a pilot study^3^ using the same inclusion criteria as this study, a significantly higher rate of treatment failure was reported among children treated with co-trimoxazole and gentamicin compared with those treated with procaine benzylpenicillin and gentamicin.

Our study shares some limitations with the trials in Africa and Bangladesh.^22^, ^23^ First, we did not mask clinicians or participants for ethical reasons;^22^ placebo injections were not judged justifiable in sick young infants. Second, families of enrolled infants most probably refused admission because their child was not perceived to be very sick.^22^, ^23^ Critical illness was a chosen exclusion criterion in the design phase because we believed treatment of very sick young infants with simplified antibiotic regimens would have been unethical. Nevertheless, 35 (1%) of 2453 trial participants died despite antimicrobial treatment and 265 (12%) of 2251 per-protocol infants did not respond to treatment, indicating a substantial level of severe illness among enrolled children. Third, concern has been raised about observer bias in assessing the soft study endpoints.^23^ Quality of clinical assessment was ensured through repeat training and supervision, and all treatment failures were confirmed independently by another study clinician, as described elsewhere.^8^, ^29^ Fourth, most infants were diagnosed clinically without availability of laboratory tests to indicate the presence or absence of infection. Since the main aim of this study was to find pragmatic solutions to scant access to antibiotic treatment for most young infants with newborn infections in low-resource settings, in environments where laboratory testing will not be available in the near future, we chose to use clinical definitions. Nevertheless, it is noteworthy that because of the absence of reliable laboratory tests to diagnose newborn infections, and the high risk of adverse outcomes, it is common practice to diagnose neonatal sepsis clinically and treat empirically with antibiotics pending laboratory results, even in high-resource hospital settings. Finally, although low case-fatality was recorded with the study treatments under stringent trial conditions and close monitoring, application of these findings in programmatic settings might not yield such impressive results when adherence with treatment might be lower. The programmatic effect of implementation of these guidelines must be assessed carefully.

Our data show that simplified antibiotic regimens with fewer injections administered closer to home by a trained health provider were as efficacious as a reference strategy comprising more penicillin-gentamicin injections for treatment of young infants with clinical severe infection whose families declined admission. Overall, this evidence supports easier to administer regimens and can inform national and international policy on the treatment of sick young infants in situations when hospital admission is not accepted.

## Figures and Tables

**Figure:**
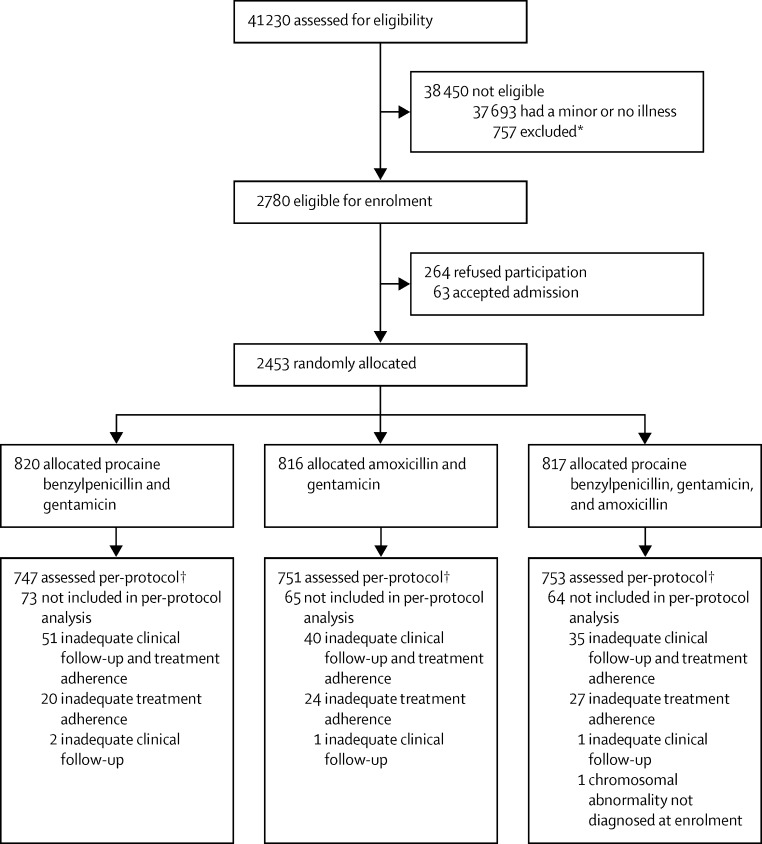
Trial flow diagram *428 children were excluded because of the presence of at least one danger sign of critical illness; 125 had low weight; 44 had a congenital malformation; 25 needed admission for a surgical reason; 87 had a history of admission in the past 2 weeks; 80 had a history of previous enrolment in the study; 55 were out of catchment area; and 106 had other comorbid conditions that needed admission. The total excluded is more than 757 because some infants fulfilled more than one criterion. †Children were included in the per-protocol analysis if they had complete or adequate clinical follow-up and complete or adequate treatment adherence (appendix p 6).

**Table 1:** Baseline characteristics

		**Procaine benzylpenicillin and gentamicin (n=820)**	**Amoxicillin and gentamicin (n=816)**	**Procaine benzylpenicillin, gentamicin, and amoxicillin (n=817)**
Age at enrolment (days)				
	0–6	361 (44%)	360 (44%)	362 (44%)
	7–59	459 (56%)	456 (56%)	455 (56%)
Sex				
	Male	465 (57%)	419 (51%)	425 (52%)
	Female	355 (43%)	397 (49%)	392 (48%)
Weight at enrolment (g)				
	<2000	62 (8%)	67 (8%)	81 (10%)
	2000–2499	145 (18%)	144 (18%)	128 (16%)
	≥28	613 (75%)	605 (74%)	608 (74%)
Weight-for-age (*Z* score)				
	<–2	303 (37%)	322 (39%)	315 (39%)
	≥–2	517 (63%)	494 (61%)	502 (61%)
Number of signs present				
	One	720 (88%)	717 (88%)	704 (86%)
	More than one	100 (12%)	99 (12%)	113 (14%)
Fever		330 (40%)	337 (41%)	348 (43%)
	In isolation	296 (36%)	303 (37%)	306 (37%)
Hypothermia		71 (9%)	71 (9%)	91 (11%)
	In isolation	43 (5%)	46 (6%)	55 (7%)
Movement only when stimulated		45 (5%)	38 (5%)	39 (5%)
	In isolation	10 (1%)	5 (1%)	4 (<1%)
Severe chest indrawing		277 (34%)	272 (33%)	269 (33%)
	In isolation	239 (29%)	238 (29%)	240 (29%)
Poor feeding or suck		210 (26%)	205 (25%)	191 (23%)
	In isolation	132 (16%)	125 (15%)	99 (12%)
Local infection		122 (15%)	110 (13%)	126 (15%)
Facility delivery*		412 (50%)	388 (48%)	423 (52%)
Maternal age (years)*		25·9 (5·5)	25·8 (5·7)	25·9 (5·5)
Maternal education (years)*		0 (0–4)	0 (0–5)	0 (0–5)

Data are number of children (%), mean (SD), or median (IQR). Signs in isolation were the only clinical sign present at enrolment.

* Data missing for place of delivery (n=1), maternal age (n=297), and maternal education (n=194).

**Table 2:** Primary and secondary treatment outcomes (per-protocol population)*

		**Procaine benzylpenicillin and gentamicin (n=747)**	**Amoxicillin and gentamicin (n=751)**	**Procaine benzylpenicillin, gentamicin, and amoxicillin (n=753)**	**Risk difference**†**(95% CI)**	**Risk difference**‡**(95% CI)**
**Primary outcome**						
Treatment failure within 7 days of enrolment		90 (12%)	76 (10%)	99 (13%)	−1·9 (−5·1 to 1·3)	1·1 (−2·3 to 4·5)
Initial reason for treatment failure						
	Death	6	4	6	..	..
	Admission	17	13	21	..	..
	Clinical deterioration	14	12	17	..	..
	New sign on or after day 3	8	11	3	..	..
	Persistence of signs at day 4	22	12	28	..	..
	Recurrence of signs on or after day 5	13	15	16	..	..
	Persistence of signs at day 8	0	0	0	..	..
	Serious adverse event	1	1	0	..	..
	Antibiotic change because of infectious comorbidity	9	8	8	..	..
**Secondary outcomes**						
Admission within 7 days of enrolment		26 (3%)	20 (3%)	30 (4%)	−0·8 (−2·6 to 0·9)	0·5 (−1·4 to 2·4)
Died within 7 days of enrolment		11 (1%)	7 (1%)	10 (1%)	−0·5 (−1·6 to 0·6)	−0·1 (−1·3 to 1·0)
Died at any time before day 15 visit		13 (2%)	9 (1%)	12 (2%)	−0·5 (−1·8 to 0·7)	−0·1 (−1·4 to 1·1)
Not classified as treatment failure with follow-up on day 11 or day 15		642 (86%)	661 (88%)	643 (85%)	..	..
Admission at any time between day 8 and day 15 visits§		6 (1%)	2 (<1%)	1 (<1%)	..	..
Died any time between day 8 and day 15 visits§		0	1 (<1%)	2 (<1%)	..	..
Non-fatal relapse at any time between day 8 and day 15 visits§		20 (3%)	9 (1%)	6 (1%)	−1·8 (−3·4 to −0·1)	−2·2 (−3·7 to −0·6)

Data are number of children (%), unless otherwise stated.

* Secondary outcomes for all randomly allocated infants are presented in the appendix (p 4).

† Difference between amoxicillin and gentamicin, and procaine benzylpenicillin and gentamicin.

‡ Difference between procaine benzylpenicillin, gentamicin, and amoxicillin, and procaine benzylpenicillin and gentamicin.

§ Denominator was children not classified as treatment failures with follow-up to day 11 or day 15.

## Panel Case definitions

**Clinical severe infection**

*Signs of clinical severe infection were defined as:*

• Movement only when stimulated
• Not feeding well on observation
• Temperature ≥38°C or <35·5°C
• Severe chest indrawing (inward chest movement with every breath in 1 min)

**Critical illness**

*Signs of critical illness were defined as:*

• Unconsciousness
• Convulsions
• Inability to feed
• Apnoea
• Inability to cry
• Cyanosis
• Bulging fontanelle
• Major congenital malformations inhibiting oral antibiotic intake
• Active bleeding needing transfusion
• Surgical conditions needing hospital referral
• Persistent vomiting (ie, vomiting after three attempts to feed the infant within 30 min, with the infant vomiting after each attempt)

## References

[1] Liu L, Oza S, Hogan D (2015). Global, regional, and national causes of child mortality in 2000–13, with projections to inform post-2015 priorities: an updated systematic analysis. Lancet.

[2] WHO (2013). Pocket book of hospital care for children: second edition—guidelines for the management of common childhood illnesses. http://www.who.int/maternal_child_adolescent/documents/child_hospital_care/en/.

[3] Zaidi AK, Tikmani SS, Warraich HJ (2012). Community-based treatment of serious bacterial infections in newborns and young infants: a randomized controlled trial assessing three antibiotic regimens. Pediatr Infect Dis J.

[4] Owais A, Sultana S, Stein AD, Bashir NH, Awaldad R, Zaidi AK (2011). Why do families of sick newborns accept hospital care? A community-based cohort study in Karachi, Pakistan. J Perinatol.

[5] Ekwochi U, Ndu IK, Osuorah CD (2015). Knowledge of danger signs in newborns and health seeking practices of mothers and care givers in Enugu state, South-East Nigeria. Ital J Pediatr.

[6] Save the Children/Saving Newborn Lives, USAID and WHO. Expert consultation on community-based approaches for neonatal sepsis management; London, UK; Sept 26–28, 2007.

[7] Baqui AH, Saha SK, Ahmed AS (2013). Safety and efficacy of simplified antibiotic regimens for outpatient treatment of serious infection in neonates and young infants 0–59 days of age in Bangladesh: design of a randomized controlled trial. Pediatr Infect Dis J.

[8] Zaidi AK, Tikmani SS, Sultana S (2013). Simplified antibiotic regimens for the management of clinically diagnosed severe infections in newborns and young infants in first-level facilities in Karachi, Pakistan: study design for an outpatient randomized controlled equivalence trial. Pediatr Infect Dis J.

[9] Tshefu A, Lokangaka A, African Neonatal Sepsis Trial (AFRINEST) group (2015). Simplified antibiotic regimens compared with injectable procaine benzylpenicillin plus gentamicin for treatment of neonates and young infants with clinical signs of possible serious bacterial infection when referral is not possible: a randomised, open-label, equivalence trial. Lancet.

[10] Baqui AH, Saha SK, Ahmed ASMNU, for the Projahnmo Study Group in Bangladesh (2015). Safety and efficacy of alternative antibiotic regimens compared with 7 day injectable procaine benzylpenicillin and gentamicin for outpatient treatment of neonates and young infants with clinical signs of severe infection when referral is not possible: a randomised, open-label, equivalence trial. Lancet Glob Health.

[11] D'Agostino RB, Massaro JM, Sullivan LM (2003). Non-inferiority trials: design concepts and issues—the encounters of academic consultants in statistics. Stat Med.

[12] Zaidi AK, Thaver D, Ali SA, Khan TA (2009). Pathogens associated with sepsis in newborns and young infants in developing countries. Pediatr Infect Dis J.

[13] Thaver D, Ali SA, Zaidi AK (2009). Antimicrobial resistance among neonatal pathogens in developing countries. Pediatr Infect Dis J.

[14] WHO Technical consultation on the use of pharmacokinetic analyses for paediatric medicine development. May 11–12, 2009. http://www.who.int/childmedicines/progress/Pharmacokinetic_June2009.pdf.

[15] Baqui AH, El-Arifeen S, Darmstadt GL, for the Projahnmo Study Group (2008). Effect of community-based newborn-care intervention package implemented through two service-delivery strategies in Sylhet district, Bangladesh: a cluster-randomised controlled trial. Lancet.

[16] Zaidi AK, Tikmani SS, Warraich HJ (2012). Community-based treatment of serious bacterial infections in newborns and young infants: a randomized controlled trial assessing three antibiotic regimens. Pediatr Infect Dis J.

[17] Cuenca AG, Wynn JL, Moldawer LL, Levy O (2013). Role of innate immunity in neonatal infection. Am J Perinatol.

[18] WHO (2012). Recommendations for management of common childhood conditions: newborn conditions, dysentery, pneumonia, oxygen use and delivery, common causes of fever, severe acute malnutrition and supportive care. http://apps.who.int/iris/bitstream/10665/44774/1/9789241502825_eng.pdf.

[19] CLSI (2010). M100-S20: performance standards for antimicrobial susceptibility testing, twentieth informational supplement.

[20] Willoughby K, Nettleton PF, Quirie M (2005). A multiplex polymerase chain reaction to detect and differentiate *Campylobacter fetus* subspecies *fetus* and *Campylobacter fetus* -species *venerealis*: use on UK isolates of *C. fetus* and other Campylobacter spp. J Appl Microbiol.

[21] WHO (2015). Managing possible serious bacterial infection in young infants when referral is not feasible: guidelines. http://www.who.int/maternal_child_adolescent/documents/bacterial-infection-infants/en/.

[22] Bhan MK, Paul VK (2015). Outpatient treatment for neonates and young infants with clinically suspected severe infection. Lancet Glob Health.

[23] Nair H, Campbell H (2015). Community management of neonatal infections. Lancet.

[24] Connell TG, Rele M, Cowley D, Buttery JP, Curtis N (2007). How reliable is a negative blood culture result? Volume of blood submitted for culture in routine practice in a children's hospital. Pediatrics.

[25] Kellogg JA, Ferrentino FL, Goodstein MH, Liss J, Shapiro SL, Bankert DA (1997). Frequency of low level bacteremia in infants from birth to two months of age. Pediatr Infect Dis J.

[26] Kotloff KL, Nataro JP, Blackwelder WC (2013). Burden and aetiology of diarrhoeal disease in infants and young children in developing countries (the Global Enteric Multicenter Study, GEMS): a prospective, case-control study. Lancet.

[27] Wagenaar JA, van Bergen MA, Blaser MJ, Tauxe RV, Newell DG, van Putten JP (2014). *Campylobacter fetus* infections in humans: exposure and disease. Clin Infect Dis.

[28] Louwen R, van Baarlen P, van Vliet AHM, van Belkum A, Hays JP, Endtz HP (2012). Campylobacter bacteremia: a rare and under-reported event?. Eur J Microbiol Immunol (Bp).

[29] Wall SN, Mazzeo CI, Adejuyigbe EA (2013). Ensuring quality in AFRINEST and SATT: clinical standardization and monitoring. Pediatr Infect Dis J.

