# Supplementary material for: Simplified antibiotic regimens for treatment of clinical severe infection in the outpatient setting when referral is not possible for young infants in Pakistan (Simplified Antibiotic Therapy Trial [SATT]): a randomised, open-label, equivalence trial
Source: Lancet Glob Health. 2016 Dec 15;5(2):e177–85. doi: 10.1016/S2214-109X(16)30335-7 (PMC5250591; doi:10.1016/S2214-109X(16)30335-7)
Supplement: Supplementary appendix [file mmc1.pdf]

THE LANCET Global Health

Supplementary appendix

This appendix formed part of the original submission and has been peer reviewed. We post it as supplied by the authors.

Supplement to: Mir F, Nisar I, Tikmani SS, et al. Simplified antibiotic regimens for treatment of clinical severe infection in the outpatient setting when referral is not possible for young infants in Pakistan (Simplified Antibiotic Therapy Trial [SATT]): a randomised, open-label, equivalence trial. *Lancet Glob Health* 2016; published online Dec 14. http://dx.doi.org/10.1016/S2214-109X(16)30335-7.

Supplementary Table 1: Study Sites Demographics

**Demographics**	**Study Sites**
Total population	274,856
Female	129,977 (47%)
Female aged 15-49 years	67,802 (25%)
Under 5 children	39,028 (14%)
Health Indicators	
Under 5 Mortality Rate (U5MR, per 1000 live births)	71.7
Infant Mortality Rate (IMR, per 1000 live births)	62
Neonatal Mortality Rate (NMR, per 1000 live births)	44.9
Maternal Mortality Rate (MMR, per 100,000 live births)	371
% coverage DPT3 (verbal report and/or card)	51.6
% with access to improved drinking water*	61
% with access to improved sanitation facilities*	49

*improved drinking water: piped household water connection located inside the user's dwelling, plot or yard
*improved sanitation facilities: 'flush toilet' connected to pit, septic tank or sewer

Supplementary Table 2: Antibiotic Dosage for Neonatal Sepsis Trials

Weight band	Amount per dose	Daily dose	Lower Limit (mg or units /kg/d)	Upper Limit (mg or units /kg/d)
**Gentamicin - desired range 4-5 mg/kg/day in 0-6 days and 5-6.5 mg/kg in 7-59 days (40mg/ml injection; single daily injection)**				
1.5-1.9 kg	0.2 ml	8 mg	4.2	5.3
2.0-2.4 kg	0.25 ml	10 mg	4.2	5.0
2.5-2.9 kg	0.3 ml	12 mg	4.1	4.8
3.0-3.9 kg	0.45	18 mg	4.6	6.0
4.0-4.9 kg	0.65	26 mg	5.3	6.5
5.0-5.9 kg	0.8	32 mg	5.4	6.4
**Procaine penicillin - desired range 40,000-60,000 units/kg/day (200,000 units/ml injection; single daily injection)**				
1.5-1.9 kg	0.4 ml	80000 units	40201	53333
2.0-2.4 kg	0.5 ml	100000 units	40161	50000
2.5-2.9 kg	0.7 ml	140000 units	48000	56000
3.0-3.9 kg	0.9 ml	180000 units	46000	60000
4.0-4.9 kg	1.1 ml	220000 units	45000	55000
5.0-5.9 kg	1.4 ml	280000 units	47500	56000
**Amoxicillin - desired range 75-100 mg/kg/day (25mg/ml (125mg/5ml); twice daily orally)***				
1.5-1.9 kg	3.0 ml	150 mg	75.4	100.0
2.0-2.4 kg	4.0 ml	200 mg	80.3	100.0
2.5-2.9 kg	5.0 ml	250 mg	83.6	100.0
3.0-3.9 kg	6.0 ml	300 mg	75.2	100.0
4.0-4.9 kg	8.0 ml	400 mg	80.2	100.0
5.0-5.9 kg	10.0 ml	500 mg	83.5	100.0

Supplementary Table 3: Blueprint for blood culture results interpretation on per protocol infants

**Pathogens traditionally 'known' to be associated with young infant sepsis in literature:**
E.coli, Klebsiella spp, Salmonella spp, Shigella spp, other Enterobacteriaceae, Pseudomonas aeruginosa, Staphylococcus aureus, Staphylococcus lugdunensis, Streptococcus pyogenes, Streptococcus agalactiae, Streptococcus pneumoniae, Burkholderia cepacia, Stenotrophomonas maltophilia, Haemophilus influenzae, Streptococcus bovis, Listeria monocytogenes, Neisseria meningitides, Acinetobacter spp, Aeromonas spp, Elizabethkingia meningoseptica, Chryseobacterium indologenes, Clostridium perfringens, Campylobacter jejuni, Campylobacter spp, Candida albicansPathogens infrequently isolated, however 'probable' causes of young infant sepsis in literature
**Pathogens infrequently isolated, however 'probable' causes of young infant sepsis in literature:**
Pseudomonas spp, Viridans streptococci (other than S.bovis), other streptococci, Enterococcus spp, Leuconostoc spp, Pediococcus spp, Clostridium spp, Brevundimonas spp, Plesiomonas shigelloides
**Contaminants (Skin flora; environmental contaminants):**
Staphylococcus spp (not aureus), Staphylococcus epidermidis, Staphylococcus saprophyticus, Corynebacterium spp, Pseudomonas stutzeri, Bacillus spp (other than Bacillus anthracis), Micrococcus spp, Diphtheroids, Propionibacterium spp, Aspergillus flavus

Supplementary Table 4: Primary and secondary treatment outcomes by treatment arm among all randomized children

		Number (%) of children		
		Arm A (N = 820)	Arm B (N = 816)	Arm C (N = 817)
Treatment failure before by D8 visit		97 (11.8%)	81 (9.9%)<br>RD = -1.9%<br>(-4.9%, 1.1%)	111 (13.6%)<br>RD = 1.8%<br>(-1.5%, 5.0%)
Initial reason for treatment failure	Death	6	5	7
	Hospitalisation	17	15	24
	Clinical deterioration	14	14	18
	New sign on/after D3	9	11	3
	Persistence of sign(s) at D4	26	12	31
	Recurrence of signs on/after D5	15	15	19
	Persistence at D8	0	0	0
	SAE	1	1	0
	Antibiotic change due to infectious co-morbidity	9	8	9
Hospitalised during first week		28 (3.4%)	22 (2.7%)<br>RD = -0.7%<br>(-2.4%, 0.9%))	36 (4.4%)<br>RD = 1.0%<br>(-0.9%, 2.9%)
Died during first week		12 (1.5%)	10 (1.2%)<br>RD = -0.2%<br>(-1.4%, 0.9%)	13 (1.6%)<br>RD = 0.1%<br>(-1.1%, 1.3%)
Died at any time before D15 follow-up		15 (1.8%)	12 (1.5%)<br>RD = -0.4%<br>(-1.6%, 0.9%)	16 (2.0%)<br>RD = 0.1%<br>(-1.2%, 1.4%)
Number of children not classified as TF with follow-up on D11 or D15		N = 668	N = 692	N = 669
Hospitalised during second week		6 (1%)	2 (<1%)	1 (<1%)
Died during second week		0	1 (<1%)	2 (<1%)
Non-fatal relapse during second week		22 (3.3%)	10 (1.5%)<br>RD = -1.8%<br>(-3.5%, -0.2%)	8 (1.2%)<br>RD = -2.1%<br>(3.7%, -0.5%)

Figure 2: Frequency of pathogens in blood cultures (81/2067) of young infants with clinical severe disease enrolled in the SATT trial, Pakistan

X axis shows absolute number of positive blood cultures (81 grew pathogens out of a total of 2067)

ł Campylobacter and related organisms include 8 C.jejuni, 1 C.upsaliensis, 1 C.coli, 3 Campylobacter spp, and 5 BACTEC positive isolates that failed to grow on solid media but were positive for the 16SrRNA conserved region of the Campylobacter/ Helicobacter/ Arcobacter complex.
Salmonella spp include 2 Salmonella spp, 2 Salmonella enterica Group D, 1 Salmonella Typhimurium, and 1 Salmonella choleraesuis-arizonae
Pseudomonads include 2 Pseudomonas spp, 1 Brevundimonas vesicularis, 2 Burkholderia cepacia, 2 Stenotrophomonas maltophilia, 1 polymicrobial culture containing Alcaligenes faecalis and Stenotrophomonas maltophilia, 1 P.aeruginosa, 1 Pseudomonas alcaligenes, 1 Pseudomonas luteola, 1 Weeksella virosa, and 1 Vibrio metschnikovii
Other streptococci include 3 Aerococcus viridans, 3 Streptococcus bovis, 1 Enterococcus faecium, and 1 Gemella morbillorum
Enterobacteriaceae include 3 E.coli, 2 Klebsiella pneumoniae, 2 Proteus mirabilis, 1 Plesiomonas shigelloides, 1 Serratia spp, 1 Enterobacter cloacae, and 2 polymicrobial cultures comprising Klebsiella pneumoniae with E.coli and Aeromonas spp
Anaerobes include 2 Clostridium perfringens, and 1 Bacteroides spp

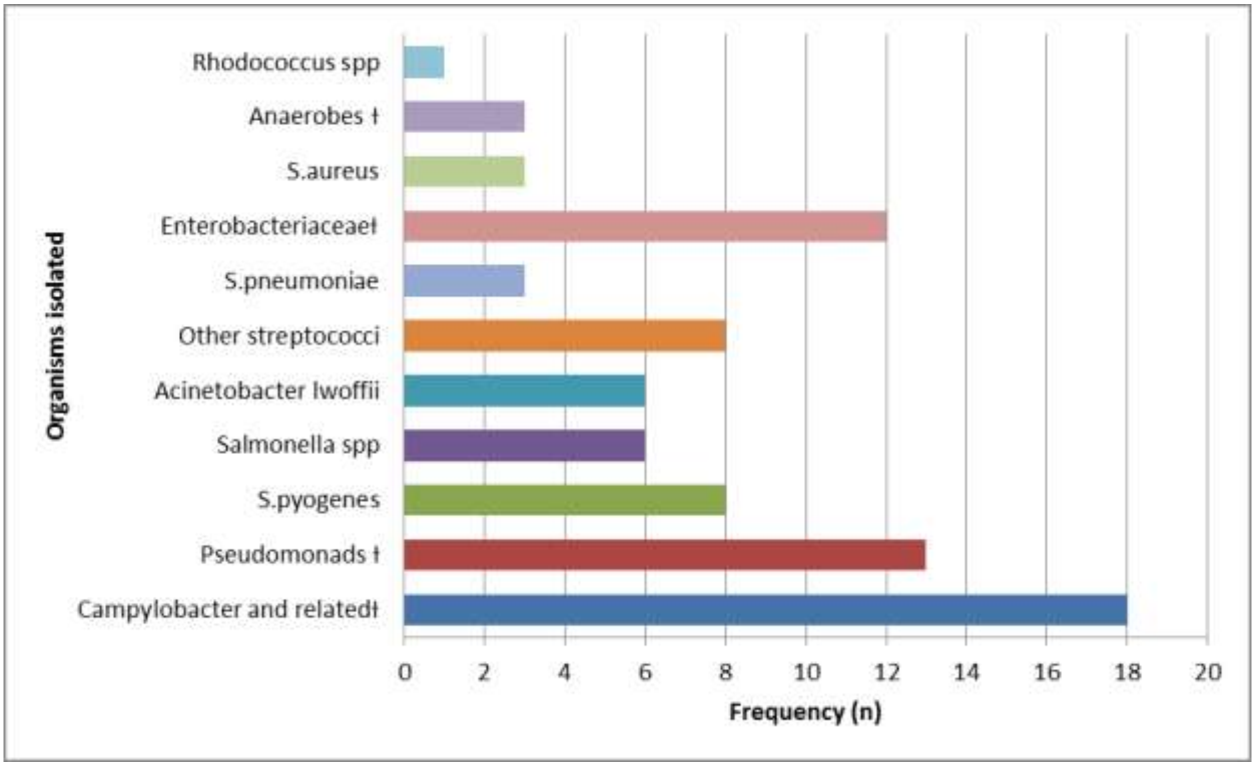

Panel 3: Per protocol analysis criteria[11]

Infants were included in the per protocol analysis provided they had received complete or partial clinical follow-up and were fully or partially treatment adherent

Adequacy of clinical follow-up

1. **Complete follow-up**: Infant had a documented treatment failure and/or clinical follow-up was completed on all 8 days.
2. **Partial follow-up**: Infant had one or more days of follow-up missing, but follow-up was completed on assessment days 2-4 and on at least one of days 5-8, and vital status on day 8 was known (vital status may be ascertained retrospectively if day 8 visit was incomplete).

Adequacy of Treatment adherence

Fully Adherent: received 100% of doses of scheduled antibiotics on all 7 days or by the time of treatment failure (TF) if TF occurred and not known to have received any other antibiotic by study or non-study physician

Partially Adherent: received 100% of scheduled antibiotics on the first 3 days of therapy or by the time of TF, **and** at least 50% of all scheduled doses of each antibiotic on days 4-7 or by the time of treatment failure; **and** did not receive any non-study injectable antibiotic before day 8 assessment (unless given due to treatment failure) or any non-study oral antibiotic on days 1-3
